# Simultaneous Electrochemical Sensing of Ultra-Trace Multiple Heavy Metals Using Metal–Organic Frameworks-Graphene Oxide Nanocomposite-Modified Electrodes

**DOI:** 10.1021/acsomega.5c07592

**Published:** 2025-11-13

**Authors:** Md Humayun Kabir, Maksudul M. Alam, Rashomi Mathanan, Elizabeth Ford, Charles C. Chusuei, Jacob A. Wolvington, Kanyon Demonbreun, William Ghann, Jamal Uddin, Mohammed Muzibur Rahman, Meser M. Ali

**Affiliations:** † Department of Chemistry and Physics, 5773 University of North Alabama, Florence, Alabama 35632, United States; ‡ InnoSense Corporation, Torrance, California 90505, United States; § 5235 Middle Tennessee State University, Murfreesboro, Tennessee 37132, United States; ∥ 1473 Coppin State University, Baltimore, Maryland 21216, United States; $ Center for Nanotechnology, Department of Natural Sciences, 1473 Coppin State University, Baltimore, Maryland 21216, United States; ⊥ Center of Excellence for Advanced Materials Research (CEAMR) & Department of Chemistry, 37848 King Abdulaziz University, Jeddah 21589, Saudi Arabia; # Department of Oncology, Karmanos Cancer Institute, Wayne State University, Detroit, Michigan 48201, United States

## Abstract

The relentless pace of industrialization has significantly exacerbated environmental pollution, with heavy-metal ions (HMIs) emerging as some of the most persistent and toxic pollutants in natural ecosystems. Growing concerns over environmental pollution have created a need for advanced sensing technologies that offer superior sensing sensitivity, selectivity, and reliability. This work reports the development of an electrochemical sensor based on a UiO-66-NH_2_(Zr) metal–organic framework (MOF)/graphene oxide (GO) nanocomposite for the simultaneous detection of HMIs in aqueous environments. Using one-pot hydrothermal synthesis, MOFs and conductive GO materials were integrated into a single nanostructure via *in situ* growth of the UiO-66-NH_2_(Zr) MOF on the GO matrix, resulting in the formation of a stable MOF/GO nanocomposite with enhanced conductivity and increased number of effective reaction sites. The amino groups (−NH_2_) on UiO-66-NH_2_(Zr) porous materials serve as adsorption sites to capture HMIs. The morphological, structural, and electrochemical properties of the UiO-66-NH_2_(Zr)-GO nanocomposite were examined by using scanning electron microscopy/energy-dispersive spectroscopy (SEM/EDS), powder X-ray diffraction (PXRD), Fourier transform infrared (FTIR) spectroscopy, cyclic voltammetry (CV), and electrochemical impedance spectroscopy (EIS). Differential pulse anodic stripping voltammetry (DPASV) was subsequently employed for the detection of heavy-metal ions over nanomolar to micromolar concentration ranges using a UiO-66-NH_2_(Zr)-GO-modified glassy carbon (GC) electrode. The electrochemical sensor developed in this study was successfully utilized for the selective and concurrent detection of multiple HMIs, namely, copper ion (Cu^2+^), cadmium ion (Cd^2+^), and lead ion (Pb^2+^) in electrolyte solution. The sensor demonstrated achieving high selectivity and sensitivity (1.30 μA μM^–1^ for Cu^2+^, 0.50 μA μM^–1^ for Cd^2+^, and 12.38 μA μM^–1^ for Pb^2+^) with low limit of detection (LOD) (0.59 ng/mL for Cu^2+^, 0.84 ng/mL for Cd^2+^, and 2.9 ng/mL for Pb^2+^), and observed ≥85% reproducibility. The sensor demonstrated excellent long-term stability and operated effectively within a temperature range of 283–313 K, enabling the simultaneous detection of multiple heavy-metal ions from small sample volumes. The developed electrochemical method can equally be employed to detect HMIs at trace (parts-per-billion (ppb)) levels in diverse environmental matrices such as lake, river, tap water, river sediments, and wastewater.

## Graphical abstract

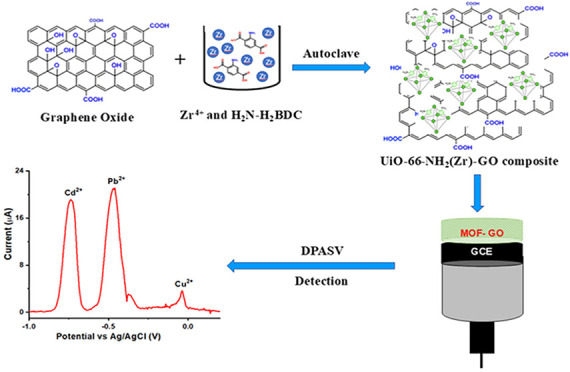

## Introduction

Environmental contamination is one of the most severe problems facing humanity today. Wastewater from industrial processes, agricultural runoff, and domestic activities has led to the release of heavy-metal ions that pose a significant threat to water resources.^1^ Even at very low concentrations, heavy metals like Cd, Pb, arsenic (As), and mercury (Hg) are highly toxic and carcinogenic. For example, mercury can damage our endocrine, nervous, and immune systems. Heavy-metal ions, such as Pb^2+^, Cu^2+^, Cd^2+^, Hg^2+^, and nickel ion (Ni^2+^), are nonbiodegradable; they persist in the environment and bioaccumulate in living organisms and humans via the food web, posing a serious risk to water resources.^2^ The World Health Organization (WHO) suggests that the concentrations of Pb^2+^, Cu^2+^, Cd^2+^, and Hg^2+^ in water should be less than 48 nM, 16 μM, 45, and 30 nM, respectively.^3^

Accurate detection of heavy-metal contaminants is essential for ensuring environmental and public safety. Developing reliable recognition systems remains a key scientific challenge. Considerable progress has been made toward identifying residual HMIs in water and other environmental samples using various analytical techniques, such as high-performance liquid chromatography (HPLC), anodic stripping voltammetry (ASV), inductively coupled plasma-atomic emission spectrometry (ICP-AES),^4^ inductively coupled plasma mass spectrometry (ICP-MS),^5^ and atomic absorption spectrophotometry (AAS),^6^ are available for detecting HMIs in water.^7–9^ However, the large-scale implementation of these analytical techniques is constrained by their high cost, operational complexity, and the need for specialized expertise. Therefore, there is an ongoing need to develop new detection strategies for HMIs in water and environmental samples that are simple to operate, rapid in response, and highly sensitive and selective.

Among various emerging approaches, electrochemical methods have gained significant attention for HMI detection because they offer rapid analysis, low operational cost, excellent sensitivity, and high potential for on-site analysis and miniaturization.^10,11^ Among various electroanalytical techniques, square-wave anodic stripping voltammetry (SWASV), differential pulse anodic stripping voltammetry (DPASV), and electrochemiluminescence are frequently utilized for the detection of HMIs. Electrochemical sensing can simultaneously detect HMIs, such as Pb^2+^, Cu^2+^, Ni^2+^, Cd^2+^, and Hg^2+^. Nevertheless, current electrochemical techniques for simultaneous HMIs detection remain limited in selectivity, sensitivity, and detection limits, warranting further optimization. Simultaneous determination of multiple HMIs in small-volume samples remains a major analytical challenge due to the possible formation of intermetallic species and competition for adsorption at active sites. Therefore, developing reliable and highly sensitive approaches for the concurrent and in situ detection of multiple HMIs in aqueous and complex matrices is of great importance. Among various sensing strategies, the design of advanced electrochemical sensors plays a key role in improving the detection performance. Metal–organic frameworks (MOFs), composed of metal nodes coordinated with multifunctional organic linkers to form highly porous architectures, have attracted significant research interest owing to their tunable structures, large surface areas, high porosity, and open metal sites.^12,13^ These characteristics render MOFs as attractive materials for gas/energy storage, catalysis, adsorption, separation, and sensing.^14–18^ Poor electronic conductivity of the MOF makes it less attractive to be used as electrode materials.

Integrating MOFs with conductive materials has proven to be an effective strategy for enhancing their electrical conductivity and structural stability.^11,19,20^ A variety of conductive materials, such as carbon-based materials, metal or metal oxide nanoparticles, conducting polymers, and graphene derivatives, have been incorporated with MOFs to significantly improve their electrochemical properties. GOs are advanced porous materials characterized by their high surface area, adjustable electrical conductivity, low density, and remarkable porosity, enabling their use in sensors, flexible electronics, adsorbents, and catalytic applications. The distinctive surface chemistry of GO, featuring hydroxyl, carboxyl, and epoxide groups, offers multiple reactive sites for interaction with −NH_2_ functionalities and metal centers of MOFs. These interactions, often through coordination with unsaturated metal sites, lead to the formation of stable MOF-GO hybrid nanostructures.^21–23^ Incorporating MOFs within the GOs framework preserves the intrinsic characteristics of both components while generating a synergistic enhancement in their overall performance. Therefore, this research endeavor is directed toward harnessing the synergistic potential of combining MOF and GO to create MOF-GO nanocomposite electrodes for developing low-volume, electrochemical sensors for the detection and identification of HMIs for ambient and extreme environmental applications. In this study, zirconium-based MOF, UiO-66-NH_2_(Zr), was selected as a sensing electrode material for the detection of HMIs because of its high thermal and chemical stability. The amino groups (−NH_2_) on UiO-66-NH_2_(Zr) porous materials can serve as adsorption sites to capture HMIs.

Among many electrochemical techniques, DPASV offers simplicity, speed, low cost, potential of low limit of detection (LOD: 1–5 ng/mL), and its detection ability for a wide selection of metal ions. It generates a peak-shaped symmetrical voltammogram. The combined effect of the MOF-GO sensor nanocomposite materials coupled with the DPASV detection will ultimately help us in the development of a highly sensitive, distinct, and reliable sensor for detecting HMIs in an aqueous environment. Electrochemical stripping analysis is a group of electroanalytical methods including DPASV is a highly effective technique for detecting trace metals, offering exceptional sensitivity through the combination of a preconcentration step and precise measurement methods, which together enhance the signal relative to background interference. This method allows for the simultaneous determination of four to six metals across a wide range of matrices, with detection limits reaching as low as 10^–10^ M—all achievable using relatively low-cost instrumentation.^24–27^

There are many applications in HMIs detection that involve the MOF-coated glassy carbon (GC) electrode.^28,29^ For example, Lu et al. developed electroanalytical techniques based on graphene aerogel and MOF UiO-66-NH_2_(Zr) composites for simultaneous monitoring of Cd^2+^, Pb^2+^, Cu^2+^, and Hg^2+^ metal ions in solutions.^20^ Qian et al. designed and prepared ZJU-27, a lanthanide metal–organic framework (Ln-MOF) to detect trace amounts of Cd^2+^ and Pb^2+^ ions in electrochemical sensors.^30^ Guo et al. have developed an electrochemical sensor based on NH_2_-MIL-53 (Cr) MOF to detect Pb^2+^ ions.^2^ Wang et al. reported the fabrication of UiO-66-NHC­(S)­NHMe/3D KSC nanocomposites designed for both the removal and electrochemical sensing of Cd^2+^, Pb^2+^, Cu^2+^, and Hg^2+^.^31^

In this work, we will investigate UiO-66-NH_2_(Zr)-GO nanocomposite as sensor materials to impart selectivity and sensitivity of the UiO-66-NH_2_(Zr)-GO@GC electrode. The Zr-MOF, UiO-66-NH_2_(Zr), was selected due to its excellent hydrophilicity, high stability to hydrolysis over a wide range of pH, simple synthesis, and extreme versatility. Additionally, the −NH_2_ groups’ strong interactions with GO surface functionalities (hydroxyl, carboxyl, and epoxide) facilitated the formation of stable UiO-66-NH_2_(Zr)-GO dispersions, ensuring uniform distribution of UiO-66-NH_2_(Zr) nanoparticles within the UiO-66-NH_2_(Zr)-GO nanocomposite. The UiO-66-NH_2_(Zr)-GO as sensor materials in conjunction with the DPASV for the detection of trace HMIs (Cd^2+^, Pb^2+^, and Cu^2+^) has been developed for the first time. The detection limits of Cd^2+^, Pb^2+^, and Cu^2+^ were found to be as low as 0.59 ng/mL.

## Materials, Methods, and Instrumentation

### Chemicals and Materials

Zirconium­(IV) chloride (ZrCl_4_, ≥99.5%), 2-aminoterephthalic acid (H_2_N–H_2_BDC, ≥99.0%), *N*,*N*-dimethylformamide (DMF), copper acetate (CH_3_COOCu), lead acetate (CH_3_COOPb), cadmium acetate (CH_3_COOCd), sodium acetate (CH_3_COONa), magnesium acetate (CH_3_COOMg), nickel acetate (CH_3_COONi), zinc acetate (CH_3_COOZn), acetic acid (CH_3_COOH), and sodium hydroxide (NaOH) were purchased from MilliporeSigma (Milwaukee, WI). All solvents and reagents were of HPLC or analytical grade and employed without further purification. Prior to use, each metal salt solution was prepared in volumetric flasks thoroughly cleaned with a 1 M nitric acid (HNO_3_) solution.

### Methods and Instrumentation

#### Morphological, Compositional, and Structural Probes

The morphological and chemical characteristics of the nanocomposite were examined using a Hitachi S-3400 scanning electron microscope equipped with an Oxford Aztec INCA X-Act energy-dispersive spectrometer (Westford, MA). The instrument was operated at an accelerating voltage of 15 kV and a magnification of 20,000×. To minimize surface charging effects and enable high-resolution imaging, the sample surface was sputter-coated with a thin layer of gold and palladium prior to analysis. Attenuated total reflectance Fourier transform infrared (ATR-FTIR) spectra were collected over the range of 4000–500 cm^–1^ using a zinc selenide (ZnSe) crystal on a Thermo Nicolet iS50 spectrophotometer (Thermo Scientific). Powder X-ray diffraction (PXRD) patterns were obtained with a Rigaku Miniflex 600 diffractometer using Cu Kα radiation (λ = 1.5406 Å), a step size of 0.02° in 2θ, and a scan rate of 2° min^–1^ over a 2θ range of 5–60° to analyze the phase of the samples.

### Synthesis of Graphene Oxide and Characterization

Graphene oxide nanosheets were synthesized from graphite powder using a modified Hummers’ method.^19,32^ Initially, 1.0 g of graphite powder was preoxidized in a mixture of 0.50 g of sodium nitrate (NaNO_3_) and 23 mL of concentrated sulfuric acid (H_2_SO_4_) under constant stirring. After 1 h, 3.0 g of potassium permanganate (KMnO_4_) was added gradually while maintaining the temperature near 5 °C using an ice bath. The reaction was subsequently continued at 35 °C for 12 h with continuous stirring. The mixture was then diluted slowly with 500 mL of deionized water under vigorous stirring, followed by the addition of 5 mL of 30% hydrogen peroxide (H_2_O_2_) to reduce any remaining KMnO_4_. The resulting suspension was purified by repeated centrifugation and filtration, first using 5% hydrochloric acid (HCl) and then deionized water until a neutral pH was achieved. Finally, the GO nanosheets were dried under vacuum for 12 h.

#### Synthesis of UiO-66-NH_2_(Zr) MOF

UiO-66-NH_2_(Zr) was synthesized using a modified hydrothermal approach.^20^ Briefly, 0.2325 g of zirconium chloride (ZrCl_4_) and 0.1810 g of 2-aminoterephthalic acid were dissolved in 50.0 mL of dimethylformamide (DMF) by ultrasonication for 2 h. The resulting solution was transferred to a 125 mL Teflon-lined stainless-steel autoclave and subjected to hydrothermal treatment at 120 °C for 48 h. After naturally cooling to room temperature, the obtained solid was washed twice with DMF and twice with methanol to remove the unreacted species. The final UiO-66-NH_2_(Zr) material was dried under vacuum at 96 °C for 24 h. UiO-66-NH_2_(Zr) features a face-centered cubic framework composed of [Zr_6_O_4_(OH)_4_] clusters interconnected by 2-aminoterephthalate linkers, forming spacious tetrahedral and octahedral cavities with amino groups oriented toward the interior pores.^33^

#### Synthesis of UiO-66-NH_2_(Zr) MOF-GO Composite

UiO-66-NH_2_(Zr)-GO nanocomposite was also prepared using a modified hydrothermal method.^20^ The reaction setup for the UiO-66-NH_2_(Zr)/GO composite synthesis is illustrated in Figure 1. In a typical procedure, 15 mg of graphene oxide was dispersed in 50.0 mL of DMF via ultrasonication for 5 h. Subsequently, 0.2315 g of zirconium tetrachloride (ZrCl_4_) and 0.1810 g of 2-aminoterephthalic acid were added to the dispersion. The mixture was further sonicated for 2 h before being transferred to a 125 mL Teflon-lined stainless-steel autoclave. Hydrothermal treatment was carried out at 120 °C for 48 h. After naturally cooling to room temperature, the resulting product was washed twice each with DMF and methanol. The final UiO-66-NH_2_(Zr)/GO nanocomposite was obtained by drying the material under vacuum at 110 °C for 24 h.

**Figure 1:**
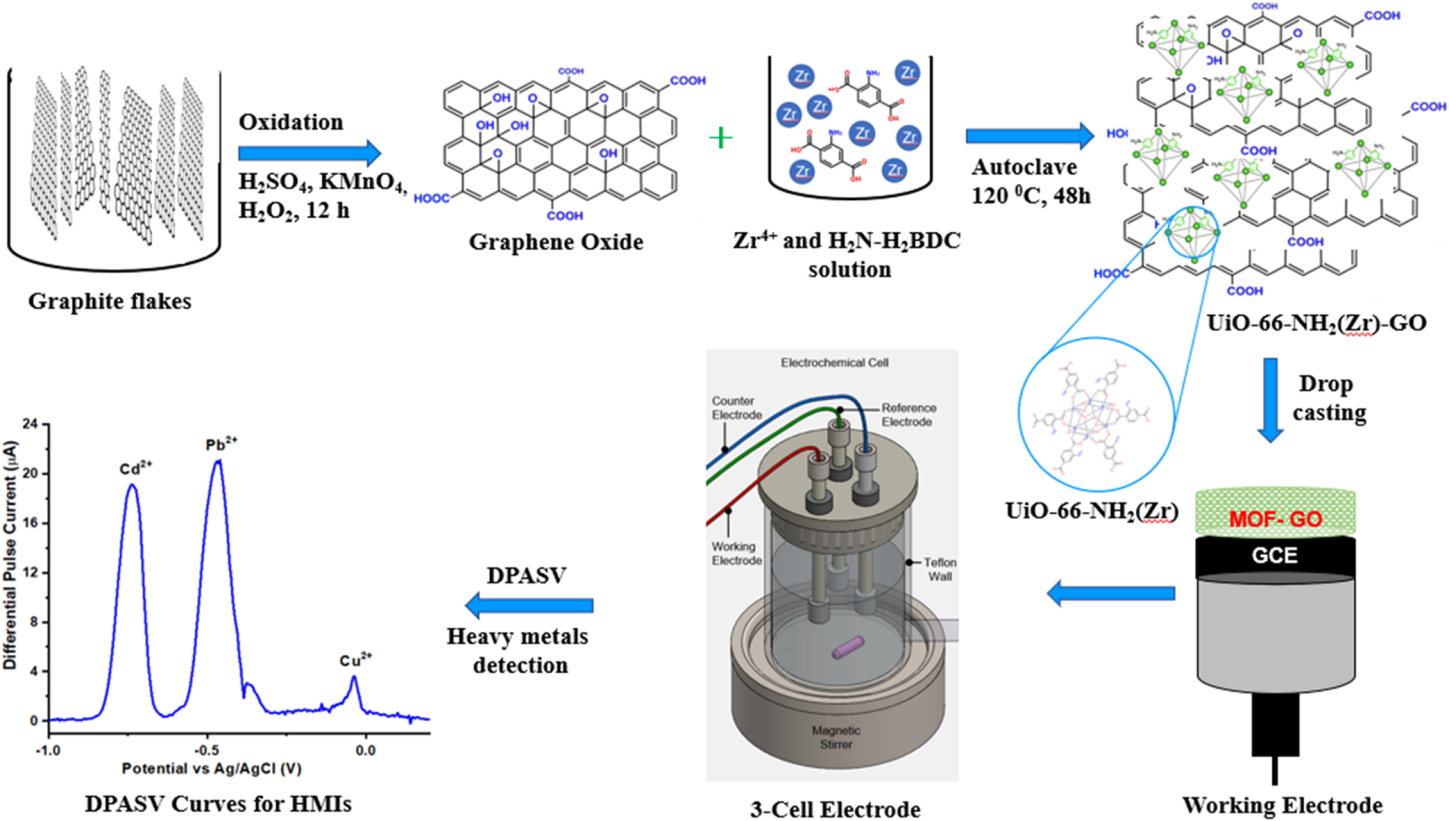
Schematic illustration of the preparation of the UiO-66-NH_2_(Zr)-GO nanocomposite-modified GCE and its application in the simultaneous sensing of multiple heavy-metal ions in aqueous solutions.

#### Preparation of UiO-66-NH_2_(Zr)-GO-Modified GC Electrodes

During the process of construction and fabrication of sensing electrodes, first, the glassy carbon electrode (GCE) was polished sequentially with 0.3 and 0.05 μm alumina slurries to eliminate surface contaminants, followed by ultrasonic cleaning in ethanol and deionized water. The synthesized UiO-66-NH_2_(Zr)/GO composite (1.5 mg) was dispersed in DMF (1 mL) under sonication for an hour. Then, 50 μL of the UiO-66-NH_2_(Zr)/GO suspension was drop-cast onto the flat surface of the GCE, forming a thin, uniform layer that was allowed to dry under ambient conditions. Afterward, a drop of Nafion solution was applied to the dried modified electrode and left to dry at room temperature. The conductive binder Nafion was employed to ensure optimal adhesion of the UiO-66-NH_2_(Zr)/GO composite on the GCE surface.

### Electrochemical Measurements: *I*–*V* Curve

First, various concentrations of metal salt solutions (Cd^2+^, Cu^2+^, and Pb^2+^) were prepared in 0.2 M acetate buffer (pH = 4.5). The Cu­(CH_3_COO)_2_, Cd­(CH_3_COO)_2_, and Pb­(CH_3_COO)_2_ salt solutions were prepared by diluting the stock salt solutions with acetate buffer to obtain concentrations ranging from 10 to 1000 nM, which served as the analytes for testing. During the accumulation step, a Teflon-coated magnetic stir bar was used to maintain the stirring at 600 rpm. To perform desired electrochemical DPASV analysis using the fabricated Zr-MOF-GO metal-sensing electrode, the classical three-electrode DPASV measurement system consists of UiO-66-NH_2_(Zr)-GO-coated GC electrode (with a surface area of 0.07 cm^2^) as a working electrode, a silver (Ag)/AgCl reference electrode, and a platinum (Pt) wire as a counter electrode. All electrochemical experiments were carried out on a Gamry electrochemical workstation. The Framework and Echem software was used for electrochemical measurements, data acquisition, and advanced processing of the results. DPASV measurements were performed with a deposition potential of −1.3 V for 400 s, a quiet time of 15 s, a stripping potential range of −1.00 to −0.50 V, a potential step of 5 mV, an amplitude of 25 mV, and a frequency of 15 Hz. After each measurement, the electrode was cleaned at 0.6 V for 180 s to remove the deposited metals. During deposition, the applied potential reduces metal ions to their metallic states over the set deposition period. Following the deposition step, stirring is halted, allowing the system to equilibrate during a 10 s quiet time. In the subsequent stripping step, the applied potential is scanned in the positive direction, causing the metal within the MOF-modified electrode to oxidize back into solution as metal ions effectively strip the metal from the electrode. The potential at which this process occurs corresponds to the redox potential of the analyte, enabling its identification from the position of the stripping peak. The peak current, in turn, reflects the amount of analyte accumulated in the electrode, which is proportional to its concentration in the solution.

## Results and Discussion

In this work, the UiO-66-NH_2_(Zr)-GO nanocomposite was employed as the recognition element of the sensor due to its synergistic structural and functional properties, making it highly suitable for HMIs detection. UiO-66-NH_2_(Zr) is a zirconium-based amino-functionalized metal–organic framework that offers an isotropic pore geometry and a well-defined, uniform particle size distribution with high monodispersity, which ensures consistent performance across sensor applications. The presence of amine functional groups on the organic linker further enhances its affinity toward heavy-metal ions through coordination interactions, hydrogen bonding, and electrostatic attractions.

The incorporation of GO into the MOF matrix significantly improves the composite’s electrical conductivity, addressing one of the primary limitations of pure MOFs in electrochemical sensing. In addition, GO provides an extended surface area and abundant oxygen-containing functional groups, contributing additional active adsorption sites that promote an effective and selective interaction with target analytes. The hybrid structure thus combines the chemical specificity of UiO-66-NH_2_(Zr) with the excellent electron transport and surface functionality of GO, leading to enhanced sensor sensitivity and response stability.

Furthermore, the nanocomposite was synthesized using a hydrothermal method, which was selected for its simplicity and cost-effectiveness. This one-pot approach allows for homogeneous mixing and strong interfacial contact between UiO-66-NH_2_(Zr) and GO, resulting in a well-integrated hybrid material with reproducible physicochemical properties. The hydrothermal process also offers precise control over crystal growth and morphology, contributing to the overall repeatability and reliability of sensor performance.

These characteristics make the UiO-66-NH_2_(Zr)-GO nanocomposite a promising platform for the development of high-performance electrochemical sensors aimed at detecting trace levels of HMIs in environmental samples.

### Powder X-ray Diffraction (PXRD) Characterization

PXRD patterns of graphene oxide, UiO-66-NH_2_(Zr), and UiO-66-NH_2_(Zr)-GO are presented in Figure 2. The diffractograms of GO show a broad diffraction peak at 2θ = 26.53° (002), which is consistent with other literature values. The presence of this peak indicates effective exfoliation of graphene layers. The synthesized UiO-66-NH_2_(Zr)-GO nanocomposite has a good crystal structure and shows the same diffraction peaks as UiO-66-NH_2_(Zr) at 2θ = 21, 30, 43, and 50°, demonstrating that the integration of GO did not change the UiO-66-NH_2_(Zr) crystal structure, which are in good agreement with that reported in the literature.^34^

**Figure 2:**
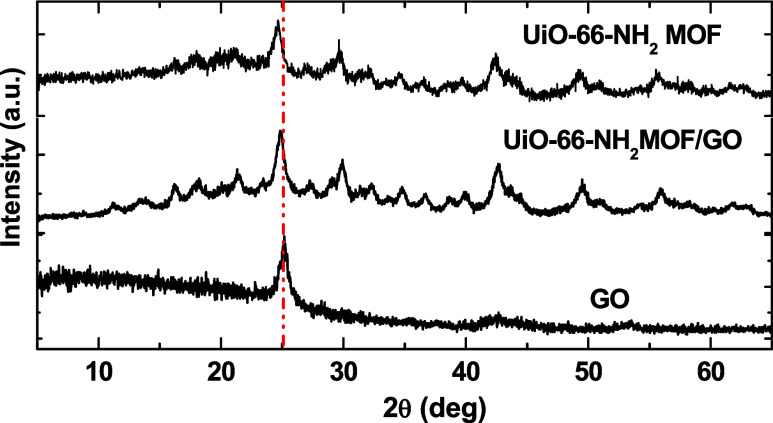
XRD patterns of GO, UiO-66-NH_2_(Zr), and the UiO-66-NH_2_(Zr)-GO nanocomposite.

### FTIR Spectroscopy Characterization

#### FTIR of GO

The synthesized GO was characterized by FTIR analysis. Its FTIR spectrum (marked in green) is shown in Figure 3. Several molecular vibration frequencies were recorded in GO specimen, which indicates that GO specimen has hydroxyl (−OH), carbonyl (C=O), and epoxy (C–O–C) groups. In GO, the peak at 1720 cm^–1^ corresponds to the carbonyl (C=O) group, while the band around 1616 cm^–1^ is attributed to the characteristic C=C stretching vibrations of the benzene ring in the graphene backbone.^19^

**Figure 3:**
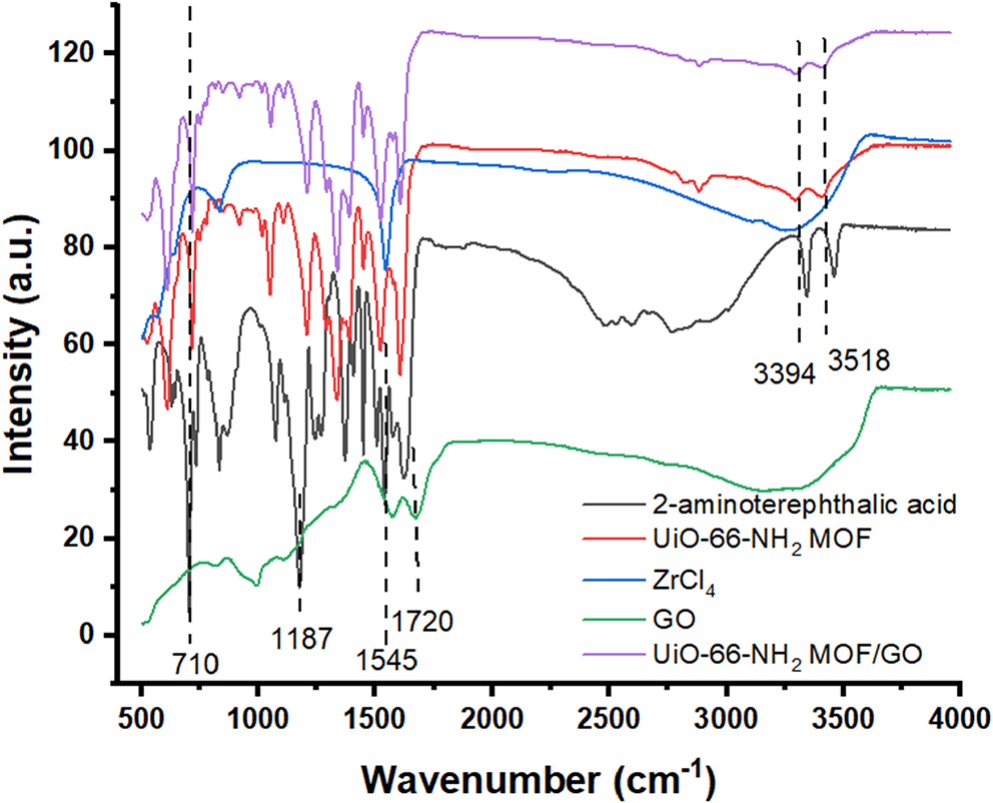
FTIR spectra of GO, zirconium chloride (ZrCl_4_), 2-aminoterephthalic acid, UiO-66-NH_2_(Zr) MOF, and UiO-66-NH_2_(Zr)-GO nanocomposite.

#### FTIR of UiO-66-NH_2_(Zr) and UiO-66-NH_2_(Zr)-GO Nanocomposite

Figure 3 also shows the FTIR spectra of UiO-66-NH_2_(Zr) and UiO-66-NH_2_(Zr)-GO nanocomposite along with their component compounds, and the N–H stretching region of the UiO-66-NH_2_(Zr) and UiO-66-NH_2_(Zr)-GO nanocomposites in the spectra. The –NH_2_ peaks are assigned as ν_sym_(NH_2_) = 3518 cm^–1^ and ν_asym_(NH_2_) = 3394 cm^–1^.^35^ The band observed at 757 cm^–1^ corresponds to the C–C stretching vibration characteristic of aromatic structures. The prominent peaks can be attributed to the skeletal vibrations of the MOF framework originating from the aromatic linker (H_2_N–H_2_BDC) and the Zr_6_O_4_(OH)_4_ nodes. The spectra of the benzene ring framework show distinct C–C vibrational bands at 1600–1565, 1500–1430 cm^–1^, and around 700 cm^–1^. Additionally, the carboxylate groups exhibit two notable features: an asymmetric stretching band appearing at 1650–1550 cm^–1^ and a weaker symmetric stretching band near 1350 cm^–1^. The lowest frequency at 570 cm^–1^ could be assigned to Zr–O stretching in the [Zr_6_O_4_(OH)_4_] clusters of UiO-66-NH_2_(Zr).^36^ In the UiO-66-NH_2_/GO composite, the characteristic peaks of GO disappeared, indicating that the oxygen-containing groups of GO interacted with the −NH_2_ groups and the open metal sites (Zr^4+^) of UiO-66-NH_2_. The FT-IR spectrum of the UiO-66-NH_2_(Zr)-GO nanocomposite was relatively unchanged, while the intensity of some peaks slightly decreased.

### SEM/EDX Characterization

The surface morphologies of the GO, UiO-66-NH_2_(Zr), and UiO-66-NH_2_(Zr)-GO electrode materials at nanometer resolution were analyzed by scanning electron microscopy (SEM). The sample was placed in a high-vacuum chamber. Various imaging parameters, including a *Z* height of 10 mm and an accelerating voltage of 30 kV, along with different detector configurations, were used to capture surface structures across multiple length scales and sensitivities. A high-energy electron beam scanned the sample surface, producing topographical images displayed on the screen.

#### SEM Characterization of UiO-66-NH_2_(Zr) and UiO-66-NH_2_(Zr)-GO Nanocomposite

The SEM image of UiO-66-NH_2_(Zr) MOF confirmed that the as-prepared UiO-66-NH_2_ MOF is crystalline and accumulated as random-intergrown morphology (Figure 4A). The UiO-66-NH_2_(Zr)-GO nanocomposite material had a higher tendency to grow from the same activation point in different directions making clumps that look like multiple smaller crystals fused with less regular shapes, as shown in Figure 4B.^20^ The GO served as the main skeleton of the composites, and UiO-66-NH_2_(Zr) crystalline nanoparticles were embedded within the GO matrix. Figure 4C,D shows EDS analysis of UiO-66-NH_2_(Zr) MOF and UiO-66-NH_2_(Zr)-GO nanocomposite that confirmed their compositions.

**Figure 4:**
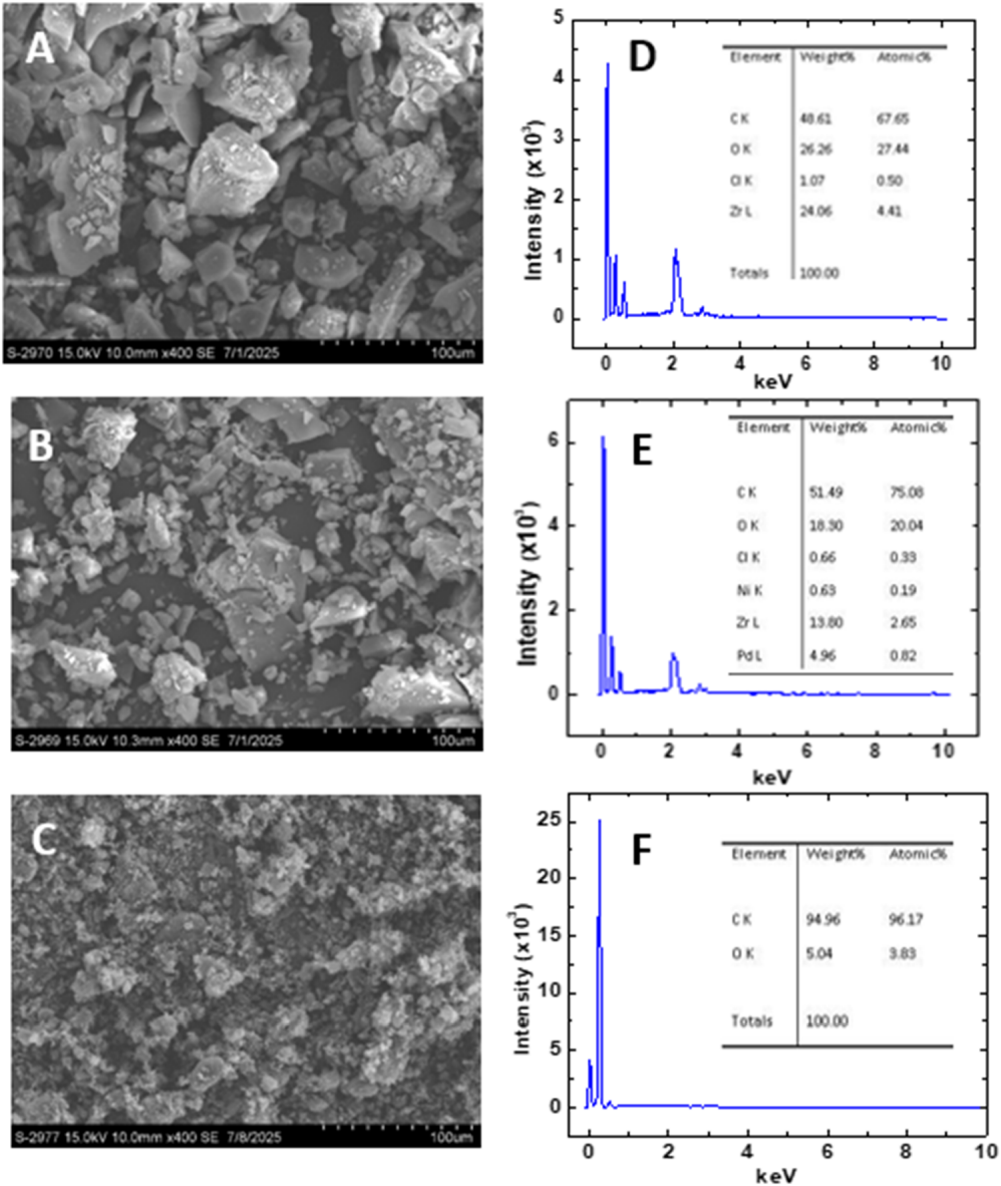
(a) SEM images showing surface morphologies of the synthesized (A) UiO-66-NH_2_(Zr) MOF, (B) UiO-66-NH_2_(Zr)-GO nanocomposite, and (C) GO. EDS images were obtained for (D) UiO-66-NH_2_(Zr) MOF, (E) UiO-66-NH_2_(Zr)-GO nanocomposite, and (F) GO.

#### SEM Characterization of GO

The surface morphologies of the synthesized GO materials at nanometer resolution were analyzed using SEM analysis. Figure 4C shows an SEM image of the synthesized GO. The surface morphology of the synthesized GO exhibits aggregated and porous structures composed of crumpled and wrinkled sheets. These folded layers form irregular clusters and a sponge-like texture, which is characteristic of exfoliated GO resulting from oxidation and exfoliation of graphite. Compared to pristine graphite, which usually presents as compact and layered platelets, GO exhibits a more disordered, rough, and expanded surface. This change in morphology confirms successful exfoliation and introduction of oxygen-containing groups. Figure 4F shows the EDS analysis of GO that confirmed the distribution of C and O in the GO nanosheets.

### Electrochemical Characterization of the UiO-66-NH_2_(Zr)-GO-Modified GC Electrodes

The electrochemical behaviors of GO-, UiO-66-NH_2_(Zr)-, and UiO-66-NH_2_(Zr)-GO-modified GC electrodes were investigated by cyclic voltammetry (CV) and electrochemical impedance spectroscopy (EIS) in a neutral electrolyte containing 0.005 M K_3_[Fe­(CN)_6_], 0.005 M K_4_[Fe­(CN)_6_], and 0.1 M KCl. The CV responses of the various electrodes, recorded at a scan rate of 50 mV s^–1^ within a potential range of −0.1 to 0.5 V, are presented in Figure 5. Compared with the bare GCE, both anodic and cathodic peak currents decreased after modification with UiO-66-NH_2_(Zr) and UiO-66-NH_2_(Zr)-GO. The redox peak separation (Δ*E*_p_) increased from 124 mV (bare GCE) to 370 mV for UiO-66-NH_2_(Zr), indicating hindered electron transfer of the [Fe­(CN)_6_]^3‑/4‑^ couple. In contrast, the Δ*E*_p_ for the UiO-66-NH_2_(Zr)-GO electrode remained comparable to that of the bare GCE, suggesting improved electron-transfer kinetics. This enhancement can be attributed to the synergistic interaction between UiO-66-NH_2_(Zr) and GO, which enhances the overall conductivity of the nanocomposite.

**Figure 5:**
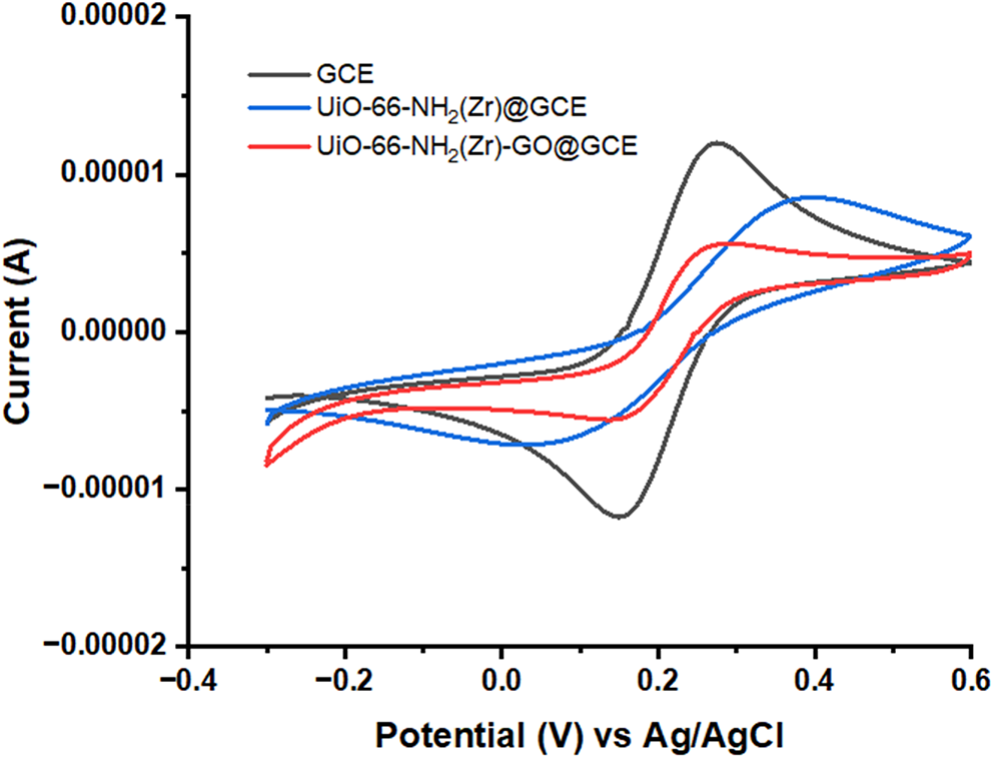
Cyclic voltammograms of GCE, UiO-66-NH_2_(Zr)@GCE, and UiO-66-NH_2_(Zr)-GO@GCE in a neutral solution containing 0.005 M K_3_[Fe­(CN)_6_], 0.005 M K_4_[Fe­(CN)_6_] and 0.1 M KCl.

The EIS was performed on GO, UiO-66-NH_2_(Zr), and UiO-66-NH_2_(Zr)-GO@GC modified electrodes and the bare GCE to investigate interfacial properties and changes in impedance. The resulting EIS curves consisted of a semicircular region at high frequencies, reflecting electron-transfer resistance, and a linear region at low frequencies, corresponding to diffusion processes. Figure 6 presents the Nyquist plots of the different electrodes. Comparison of the semicircle diameters indicates that the electron-transfer resistance of UiO-66-NH_2_/GCE is significantly higher than that of the bare GCE, suggesting that the incorporation of UiO-66-NH_2_(Zr) increases resistance and reduces electrode conductivity. In contrast, modification of the bare GCE with the UiO-66-NH_2_(Zr)/GO composite resulted in a lower electron-transfer resistance than UiO-66-NH_2_/GCE, indicating that the presence of GO facilitates electron transfer. These findings confirm the successful integration of GO with UiO-66-NH_2_(Zr) and its effectiveness in enhancing the charge-transfer kinetics of the composite.

**Figure 6:**
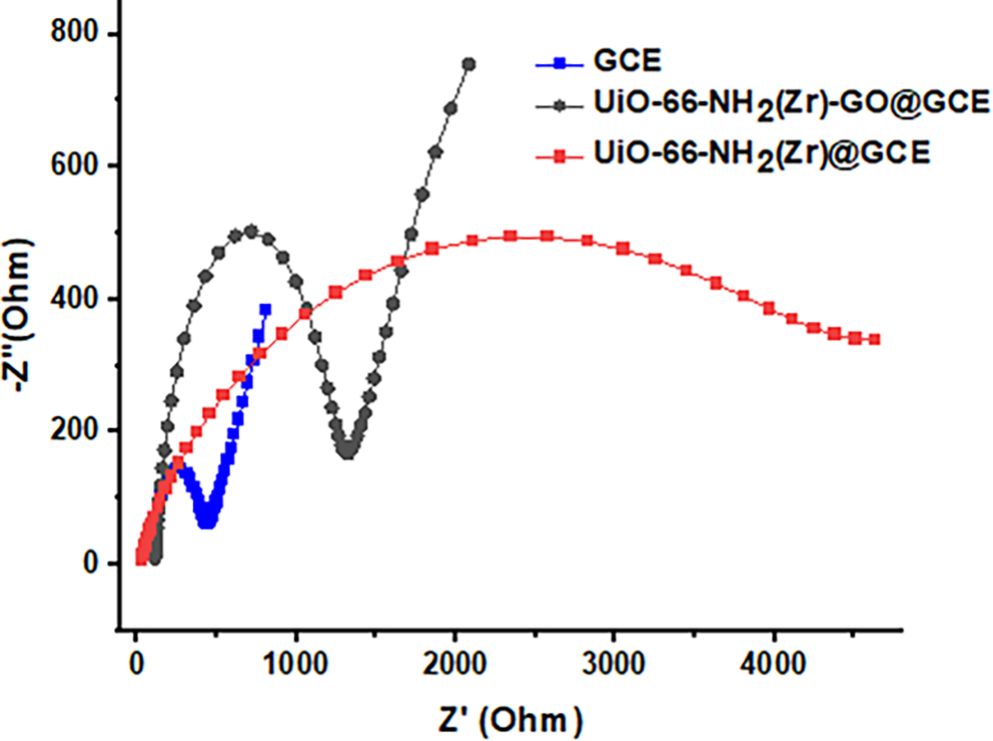
Electrochemical impedance spectra. Nyquist plots of the bare GCE, UiO-66-NH_2_(Zr)@GCE, and UiO-66-NH_2_(Zr)-GO@GCE in an electrolyte solution containing 0.005 M K_3_[Fe­(CN)_6_], 0.005 M K_4_[Fe­(CN)_6_], and 0.1 M KCl.

### Optimization of Analytical Parameters for Simultaneous Detection of Heavy-Metal Ions

To achieve optimal detection efficiency using the UiO-66-NH_2_(Zr)-GO nanocomposite-coated GCE, key experimental parameters, including solution pH, deposition potential, and deposition time, were systematically optimized for the simultaneous detection of Cd^2+^, Pb^2+^, and Cu^2+^ ions.

#### Effect of pH

Solution pH significantly influences the electrochemical response of the heavy-metal ions. In this study, the impact of pH on the peak current was investigated over the pH range of pH 3.0–6.0. The peak currents for Cd^2+^, Pb^2+^, and Cu^2+^ increased with pH from 4.0 to 4.5, reaching a maximum at pH 4.5. Beyond this point, the currents declined. The reduced response at low pH (<4.5) is attributed to the protonation of amino hydrophilic groups on UiO-66-NH_2_(Zr), which weakens metal ion adsorption.^37^ At higher pH levels (5.0 and 6.0), hydrolysis of metal ions likely inhibits their accumulation on the electrode surface. Based on these findings, pH 4.5 was selected as the optimal value for further analysis.

#### Effect of Deposition Potential

Deposition potential plays a critical role in enhancing the sensitivity. Potentials ranging from −1.0 to −1.3 V were examined. As the deposition potential became more negative, the peak currents of Cd^2+^ and Pb^2+^ increased sharply, indicating enhanced reduction and accumulation of metal ions. The highest sensitivity was observed at −1.2 V, which was chosen as the optimal deposition potential.

#### Effect of Deposition Time

Deposition time affects the accumulation of heavy-metal ions on the electrode surface, thus influencing the current response. The effect was studied over a range of 100–500 s. Peak currents increased with longer deposition times, reaching a maximum of 400 s. Beyond this, signal changes were minimal, likely due to electrode surface saturation or competition among ions for active sites. To balance the sensitivity with analytical efficiency, a deposition time of 400 s was selected for subsequent measurements.

#### Selective Determination of Individual of Heavy-Metal Ions

DPASV measurements were carried out using the UiO-66-NH_2_(Zr)-GO@GC electrode in acetate buffer (pH 4.5) with Cd^2+^ concentrations ranging from 10 nM to 1.0 μM. The peak current exhibited a steady increase with rising Cd^2+^ concentration (Figure 7A), which can be attributed to enhanced Cd^2+^ accumulation on the electrode surface at higher ion levels. A linear dependence between the differential pulse peak current (I) and Cd^2+^ concentration was obtained across the 10 nM to 1.0 μM range (Figure 7B), where *I* (μA) = 7.9786 × *C*_Cd_^2+^ (μM) – 4.2715 with a correlation coefficient of *R* ^2^ = 0.9522. The LOD for Cd^2+^ was found to be 7.5 nM (0.84 ng/mL). The limit of detection is calculated as^38^

1 $$\mathrm{LOD}=3S_{b}/m$$

where *S*_b_ is the standard deviation obtained from the blank responses and *m* corresponds to the slope of the calibration plot.

**Figure 7:**
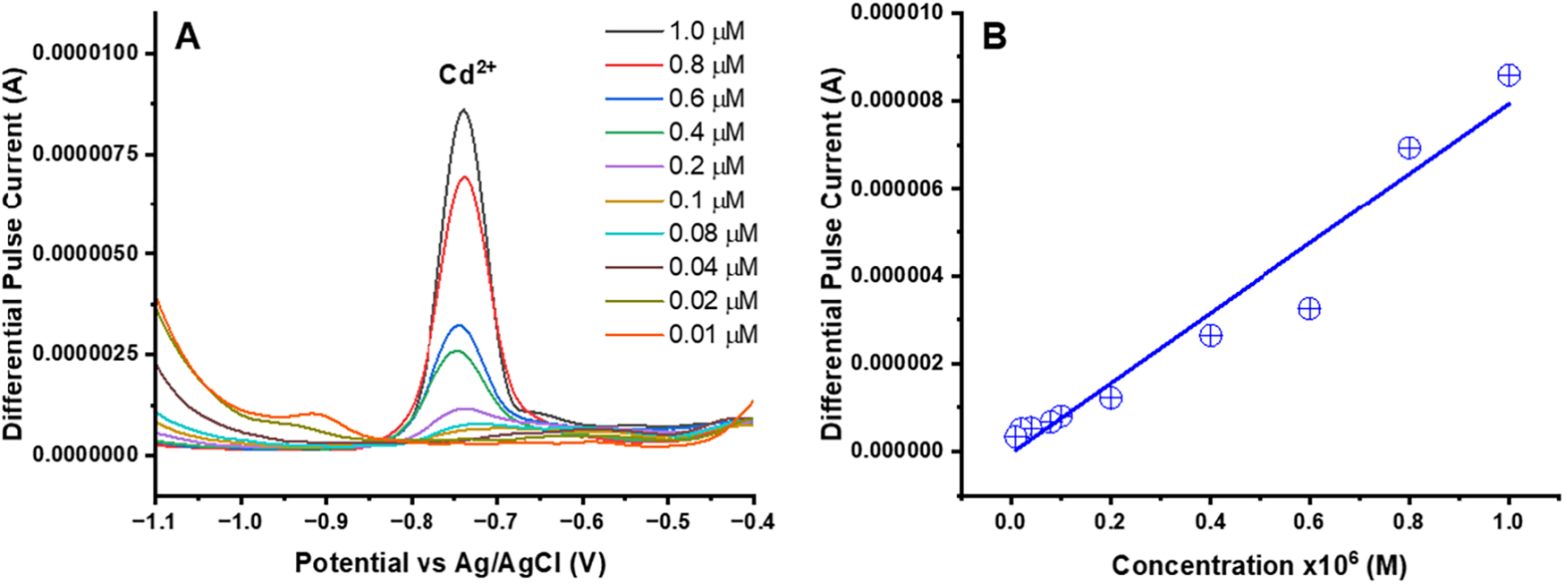
DPASV measurements of the UiO-66-NH_2_(Zr)-GO@GC electrode for Cd^2^. (A) DPASV signal for a single UiO-66-NH_2_(Zr)-GO nanocomposite-coated GC electrode for Cd^2+^ and (B) a linear calibration curve exhibiting the relationship between the output signal of the sensor and the concentration of Cd^2+^. The experiment parameters were as follows: pH: 4.5; optimized deposition potential: −1.2 V (*E* vs Ag/AgCl); deposition time: 400 s.

Using a similar DPASV measurement procedure, Cu^2+^ was also detected from its solution with concentrations ranging from 0.01 to 1.0 μM using the UiO-66-NH_2_(Zr)-GO electrode-based sensor devices (Figure 8A). A linear relationship between the peak current (*I*_peak_) and the Cu^2+^ concentration (*C*_Cu_^2+^) in the above range, which is *I*_peak_ (μA) = 3.2074 × *C*_Cu_^2+^ (μM) + 0.3725 with a correlation coefficient of *R* ^2^ = 0.9806, is given in Figure 8B. The LOD of the sensor device for the Cu^2+^ was found to be 9.36 nM (0.59 ng/mL).

**Figure 8:**
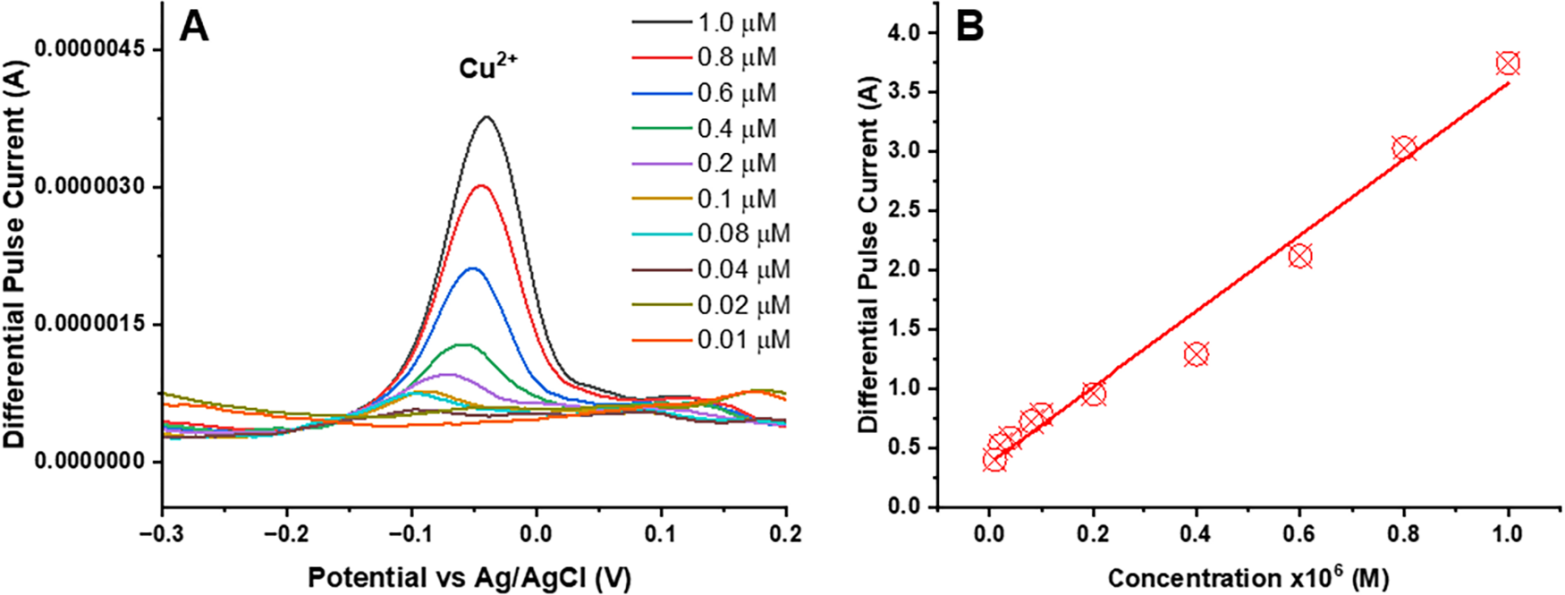
DPASV measurements of the UiO-66-NH_2_(Zr)-GO@GC electrode for Cu^2+^: (A) DPASV signal for a single UiO-66-NH_2_(Zr)-GO nanocomposite-coated GC electrode for Cu^2+^ and (B) a linear calibration curve exhibiting the relationship between the output signal of the sensor and the concentration of Cu^2+^. The experiment parameters were as follows: scan rate 25 mV s^–1^, pH: 4.5; optimized deposition potential: −1.2 V (*E* vs Ag/AgCl).

Similarly, Pb^2+^ was detected by UiO-66-NH_2_(Zr)-GO@GC electrode-based sensor devices (Figure 9A). A linear relationship between the peak current (*I*_peak_) and the Pb^2+^ concentration (*C*_Pb_^2+^) was observed, which is *I*_peak_ (μA) = 6.3813 × *C*_Pb_^2+^ (μM) −5.0180 with a correlation coefficient of *R* ^2^ = 0.9112 (Figure 9B). The LOD of the developed electrochemical sensor for Pb^2+^ was found to be 14.1 nM (2.9 ng/mL). As can be seen from Figure 9 A, double stripping peaks have been observed for Pb^2+^. Within the Pb^2+^ concentration range of 40–100 nM, a weak differential pulse voltammetry (DPV) stripping signal appeared at potentials between −0.44 and −0.42 V. When the Pb^2+^ concentration exceeded 40 nM, an additional stripping peak emerged near −0.52 V, and its intensity increased progressively with further concentration rise. The appearance of the weak second peak at elevated [Pb^2+^] levels is likely associated with alterations in the deposition morphology, possibly comprising both thin films and nanoparticles.^39,40^ It is worth mentioning in this study that Cu^2+^, Cd^2+^, and Pb^2+^ were determined individually under optimized conditions, and the stripping peak current increased consistently with ion concentration. However, the calibration curves did not exhibit ideal linearity. This deviation is commonly observed in anodic stripping voltammetry and may arise from several factors: (i) partial saturation or heterogeneity of active adsorption sites on the UiO-66-NH_2_/GO-modified electrode surface at higher analyte concentrations; (ii) diffusion or kinetic limitations becoming more significant as concentration increases; (iii) minor electrode surface restructuring or partial fouling during repeated deposition/stripping cycles; and (iv) background and capacitive currents that can introduce slight curvature in the calibration plots. Although perfect linearity was not obtained, the overall trend clearly demonstrates a concentration-dependent increase in the signal, enabling reliable quantitative detection.

**Figure 9:**
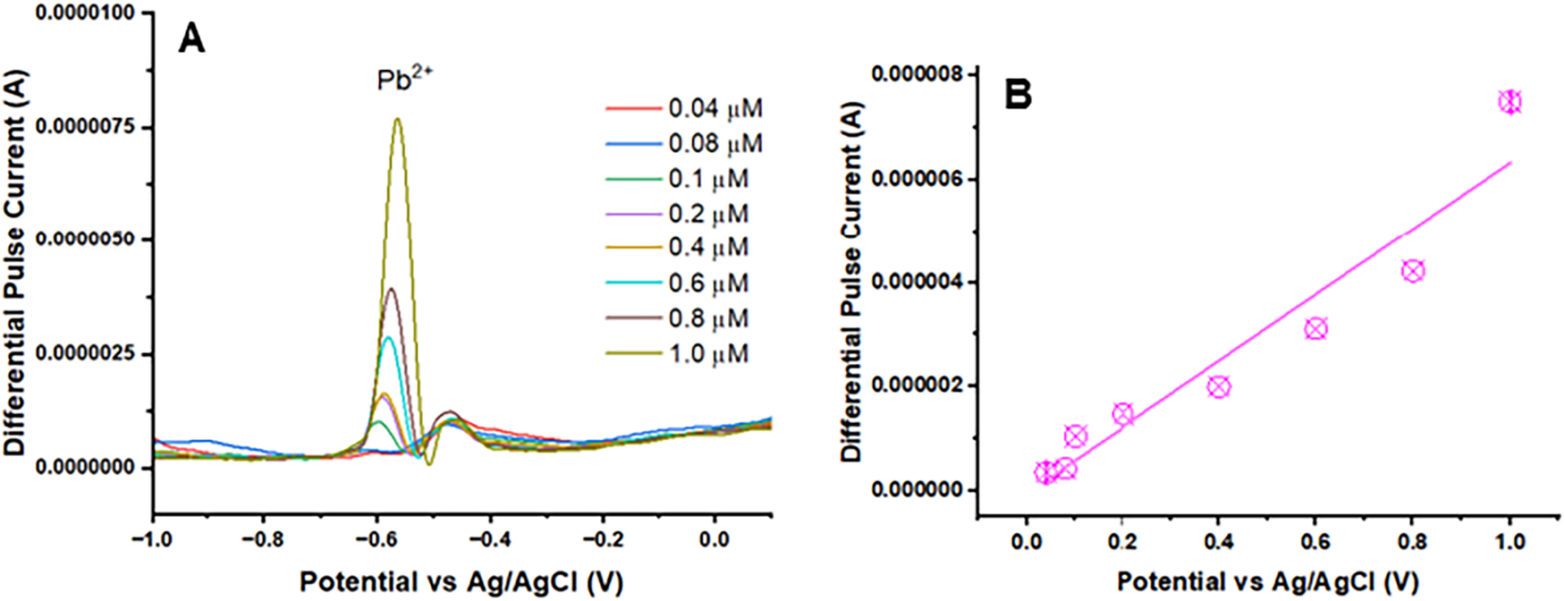
DPASV measurements of the UiO-66-NH_2_(Zr)-GO@GC electrode for Pb^2+^: (A) DPASV signal for a single UiO-66-NH_2_(Zr)-GO nanocomposite-coated GC electrode for Pb^2+^ and (B) a linear calibration curve exhibiting the relationship between the output signal of the sensor and the concentration of Pb^2+^. The experiment parameters were as follows: scan rate 25 mV s^–1^, pH: 4.5; optimized deposition potential: −1.2 V (E vs Ag/AgCl).

#### Concurrent Determination of Multiple Heavy-Metal Ions

DPASV measurements of UiO-66-NH_2_(Zr)-GO@GC electrode-based sensor devices were performed in an acetate buffer solution (pH = 4.5) containing various concentrations of mixed metal salts (Cd^2+^, Pb^2+^, and Cu^2+^) ranging from 10 nM to 1.0 μM. The DPASV peak currents corresponding to each metal salt increased progressively with higher-metal ion concentrations (Figure 10A). The calibration plots constructed for Cd^2+^, Pb^2+^, and Cu^2+^ demonstrated strong linear correlations between the measured current responses and ion concentrations (Figure 10B). The respective linear regression equations for Cd^2+^, Pb^2+^, and Cu^2+^ were *I*_peak_ (μA) = 0.4981 × *C*_Cd_^2+^ (μM) + 0.0149 (*R* ^2^ = 0.9808), *I*_peak_ (μA) = 0.3238 × *C*_Pb_^2+^ (μM) + 0.0439 (*R* ^2^ = 0.9729), and *I*_peak_ (μA) = 1.2991 × *C*_Cu_^2+^ (μM) + 0.0673 (*R* ^2^ = 0.9451), respectively. The LODs for each metal ion were found to be 60 nM (6.77 ng/mL) for Cd^2+^, 0.13 μM (27.76 ng/mL) for Pb^2+^, and 23 nM (1.46 ng/mL) for Cu^2+^. The LODs of all of the heavy metals are summarized in Table 1. The sensitivities of the UiO-66-NH_2_(Zr)-GO@GC electrode for the analyzed HMIs are related to the slopes of the corresponding fitted straight lines. The sensitivities for Cu^2+^, Cd^2+^, and Pb^2+^ are 1.30, 0.50, and 12.38 μA μM^–1^, respectively.

**Figure 10:**
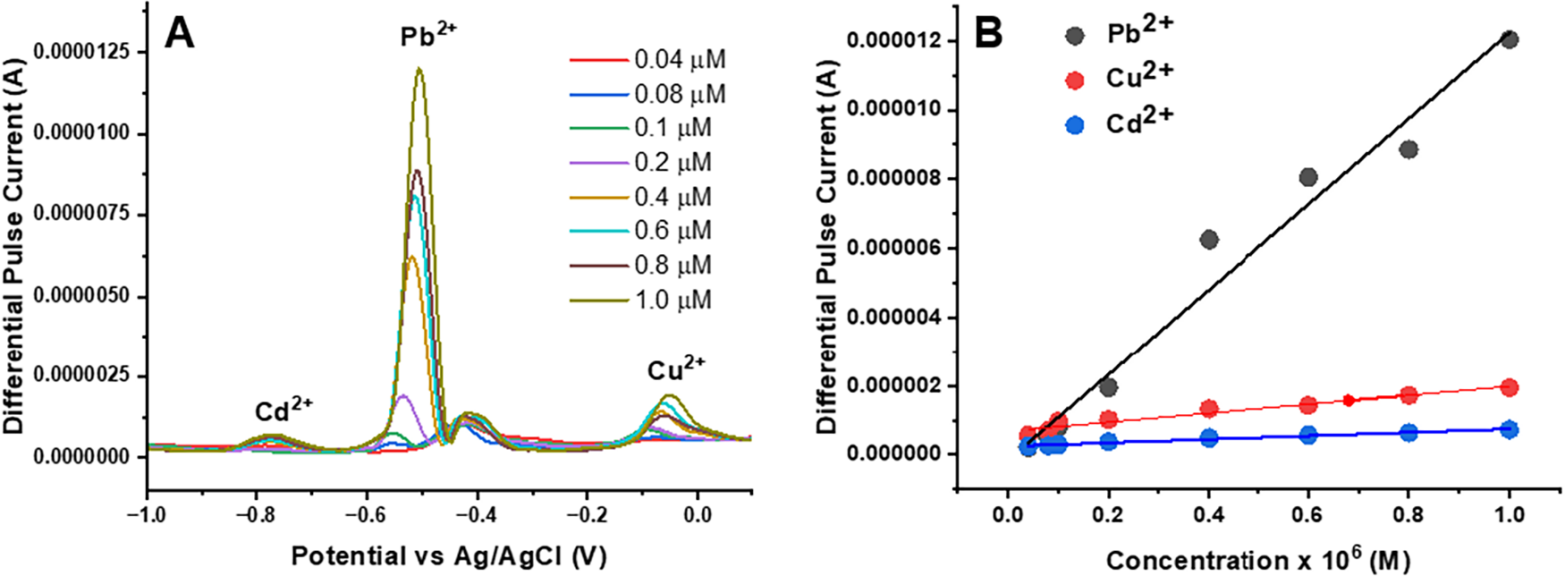
DPASV measurements of the UiO-66-NH_2_(Zr)-GO nanocomposite-coated GC electrode for a mixture of metal salts: (A) DPASV signal for a single UiO-66-NH_2_(Zr)-GO nanocomposite electrode after adding different concentrations of Cd^2+^, Pb^2+^, and Cu^2+^ in acetate buffer solution and (B) linear calibration curves exhibiting the relationship between the output signal of the sensor and the concentration of Cd^2+^, Pb^2+^, and Cu^2+^. The experimental parameters were as follows: pH: 4.5; optimized deposition potential: −1.2 V (*E* vs Ag/AgCl); deposition time: 400 s.

**Table 1:** Comparison of the Linear Range and Detection Limit of the Proposed UiO-66-NH_2_(Zr)-GO@GCE Electrochemical Sensor for Simultaneous Determination of Heavy-Metal Ions with Those of Various Other Electrochemical Sensors

electrode	method	heavy-metal ion	linear range (μmol L^–1^)	detection limit (nmol L^–1^)	refs
GA-UiO-66-NH_2_/GCE	DPSV	Cd^2+^	0.01–1.5	9.0	^20^
		Pb^2+^	0.001–2.0	1.0	
		Cu^2+^	0.01–1.6	8.0	
		Hg^2+^	0.001–2.2	0.9	
MIL-100(Cr)/GCE	DPSV	Cd^2+^	0–10	44	^27^
		Pb^2+^	0–10	48	
		Cu^2+^	0–10	11	
		Hg^2+^	0–10	8.8	
UiO-66-NHC(S)NHMe/3D-KSC	SWASV	Cd^2+^	0.038–8.0	12.5	^31^
		Pb^2+^	0.037–8.0	12.4	
		Cu^2+^	0.033–8.0	11.1	
		Hg^2+^	0.028–8.0	9.4	
g-C_3_N_4_/CNT/NH_2_-MIL-88(Fe)	SWSV	Cd^2+^	0.12–6.0	39.6	^3^
		Pb^2+^	0.02–6.0	7.6	
		Cu^2+^	0.04–6.0	11.9	
		Hg^2+^	0.03–6.0	9.6	
UiO-66-NH_2_(Zr)-GO/GCE	DPASV	Cd^2+^	0.01–1.0	60.2	this work
		Pb^2+^	0.01–1.0	92.6	
		Cu^2+^	0.01–1.0	23.0	

It is worth mentioning that we selected the UiO-66-NH_2_/GO composite following preliminary screening of several other MOFs/graphene composites, including MOF-808, UiO-66-NH_2_–rGO (reduced graphene oxide), UiO-66-NH_2_–GA (graphene aerogel), and UiO-66-NH_2_–MWCNT. Among these, the UiO-66-NH_2_–GO composite electrode exhibited better performance in terms of sensitivity, selectivity, and LOD. These results strongly suggest that the integration of the UiO-66-NH_2_ and GO imparts favorable interfacial interactions and synergistic effects—such as greater exposure of active sites and improved structural stability—that are not observed to the same extent in their individual components as well as in MOF-808, UiO-66-NH_2_–rGO, UiO-66-NH_2_–GA, and UiO-66-NH_2_–MWCNT composites. In Table 1, the UiO-66-NH_2_/GO system compares favorably with previously reported MOF-graphene-based sensors. These comparisons underscore the novelty and practical significance of integrating UiO-66-NH_2_ with GO—both in enhancing sensing metrics and in enabling broader applicability.

#### Repeatability and Reproducibility of Sensor Devices for Detecting Metal Salts

The repeatability and reproducibility of UiO-66-NH_2_(Zr)-GO@GC electrode-based sensor devices were investigated for detecting Pb^2+^ ion in an acetate buffer solution containing 1.0 μM Pb^2+^ by using the DPSV method. Three new UiO-66-NH_2_(Zr)-GO composite electrodes were prepared and applied to investigate repeatability and reproducibility (each electrode was tested six times). Figure 11A shows DPASV signal graphs for a UiO-66-NH_2_(Zr)-GO@GC electrode-based sensor device, exhibiting that the UiO-66-NH_2_(Zr)-GO@GC electrode-based sensor device has good measurement stability toward detecting Pb^2+^. Three more UiO-66-NH_2_(Zr)-GO@GC electrodes were tested (each electrode was evaluated six times) under identical test conditions to further confirm the performance reproducibility of the sensor devices. Figure 11B shows the excellent repeatability/reproducibility of sensor devices evaluated for three UiO-66-NH_2_(Zr)-GO@GC electrodes.

**Figure 11:**
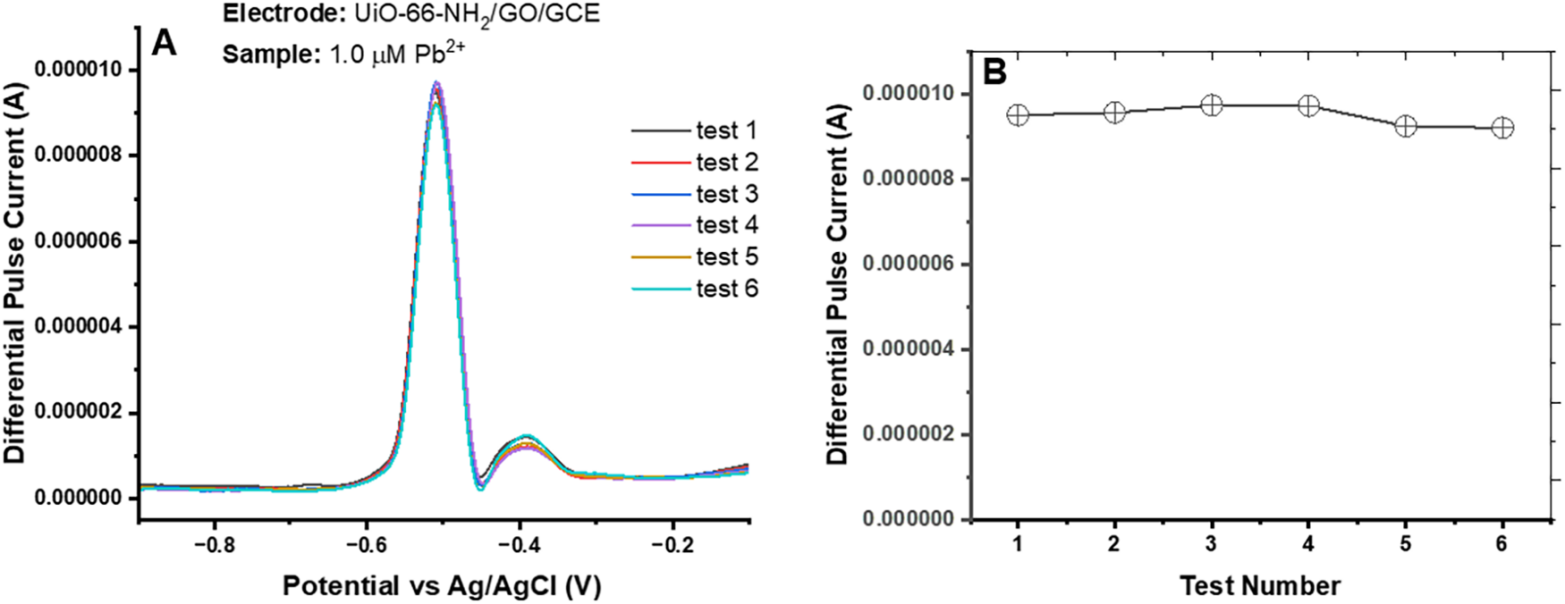
Retention/repeatability and reproducibility test of UiO-66-NH_2_(Zr)-GO nanocomposite-coated GC electrode-based sensor devices. (A) DPASV signals (repeated six times) for a single UiO-66-NH_2_(Zr)-GO nanocomposite decorated GC electrode-based sensor device and (B) repeatability/reproducibility plot of sensor devices evaluated for three UiO-66-NH_2_(Zr)-GO nanocomposite-coated GC electrodes (each electrode was tested six times). The experiment parameters were as follows: pH: 4.5; optimized deposition potential: −1.2 V (*E* vs Ag/AgCl); deposition time = 250 s.

#### Longevity Testing (Long-Term Reusability Assessment) of Sensor Devices

We assessed factors such as long-term reusability, chemical stability, and potential interference from other ions present in the sample matrix. The longevity testing (long-term reusability assessment) of UiO-66-NH_2_(Zr)-GO@GC electrode-based sensor devices for metal ions detection was investigated in an acetate buffer solution containing 0.8 μM concentration of a mixture of metal salts containing Pb^2+^, Cu^2+^, Cd^2+^, and Fe^2+^ by using the DPASV method. Figure 11 shows DPASV signal graphs of UiO-66-NH_2_(Zr)-GO@GC electrode-based sensor devices taken over a period of 6 days for evaluating their long-term reusability for simultaneous detection of multiple metal ions from a mixed solution of metal salts. These results suggest that sensor devices are highly reproducible, repeatable, and reusable for a long period of time for simultaneous quantification of multiple HMIs.

#### The Interaction Mechanism of the HMIs with the GO and MOF in the UiO-66-NH_2_(Zr)-GO Composite

The interaction mechanisms of heavy-metal ions (HMIs) such as Cu^2+^, Pb^2+^, and Cd^2+^ with MOFs and GO have been reported in prior studies, and our findings are consistent with these observations. Specifically, the UiO-66-NH_2_ MOF contains coordinatively unsaturated Zr^4+^ metal sites and free −COOH/–COO^–^ functional groups that serve as key active sites for the adsorption process.^41,42^ According to the hard–soft acid–base (HSAB) theory, Cu^2+^, Pb^2+^, and Cd^2+^ behave as borderline to soft acids, whereas the −COO^–^ groups act as soft bases, enabling strong acid–base coordination interactions. In addition, the amino (−NH_2_) functionalities of UiO-66-NH_2_ can provide supplementary binding sites through electrostatic attraction, hydrogen bonding, or coordination, further enhancing metal ion adsorption. For example, Wang et al. demonstrated that UiO-66-NH_2_ effectively adsorbs Pb^2+^ and Cd^2+^ ions via coordination with −COOH and −NH_2_ groups.^43^

On the other hand, GO contributes abundant oxygenated functional groups, such as −OH, −COOH, and epoxy groups, which interact with HMIs primarily through complexation, electrostatic interactions, and in some cases π–cation interactions with the delocalized π-electron system of graphene sheets. For instance, Ni et al. reported that HMIs bind strongly to carboxyl and hydroxyl sites on GO, enhancing both adsorption and stability.^44^ This dual contribution creates a synergistic effect in the MOF–GO composite: the MOF offers a high surface area and well-defined coordination sites, while GO enhances electron transfer, dispersibility, and additional adsorption functionality.

Taken together, these reports suggest that in our UiO-66-NH_2_–GO composite, the MOF provides well-defined coordination and functional binding sites, while GO introduces complementary oxygenated groups and enhanced electron-transfer pathways. This dual contribution creates multiple cooperative binding mechanisms for Cu^2+^, Pb^2+^, and Cd^2+^, which directly explain the high sensitivity and selectivity observed in our sensing platform.

XPS is a powerful technique to probe oxidation states, surface composition, and possible coordination environments of the MOF before and after exposure to heavy-metal ions (HMIs). XPS was not employed in this work to directly verify the binding interactions and electron density changes associated with HMI adsorption on the MOFs. However, the adsorption mechanisms were inferred through complementary characterization and analysis, which provides consistent evidence of HMI–MOF interactions. Furthermore, prior studies have established that XPS can effectively reveal the coordination environments of MOFs before and after HMI exposure, supporting the plausibility of the mechanisms proposed in our study.^31,45–48^

#### Temperature-Dependent Testing and Evaluation of Sensor Device Performance

Figure 12(A) shows DPSV signal graphs of UiO-66-NH_2_(Zr)-GO@GC electrode-based sensor devices for a 0.8 μM Pb^2+^ ion at various temperatures ranging from 283 to 313 K. The observed DPASV graphs show that the sensor signals for detecting Pb^2+^ ion are strong and changed significantly depending on the temperature of the metal salt solution being analyzed (Figure 12B). Essentially, the intensity of the DPASV signal varies with temperature, providing information about the reaction kinetics and thermodynamics involved in the redox process being studied. Increasing the temperature of the metal salt solution leads to a higher DPASV peak current, as the increased kinetic energy of the molecules facilitates faster electron-transfer reactions at the electrode surface. Higher temperatures also result in faster diffusion rates of the metal ions toward the sensing electrode, further enhancing the signal (current) response of the sensor devices. These results suggest that the sensor devices are capable of operating in a practical temperature range.

**Figure 12:**
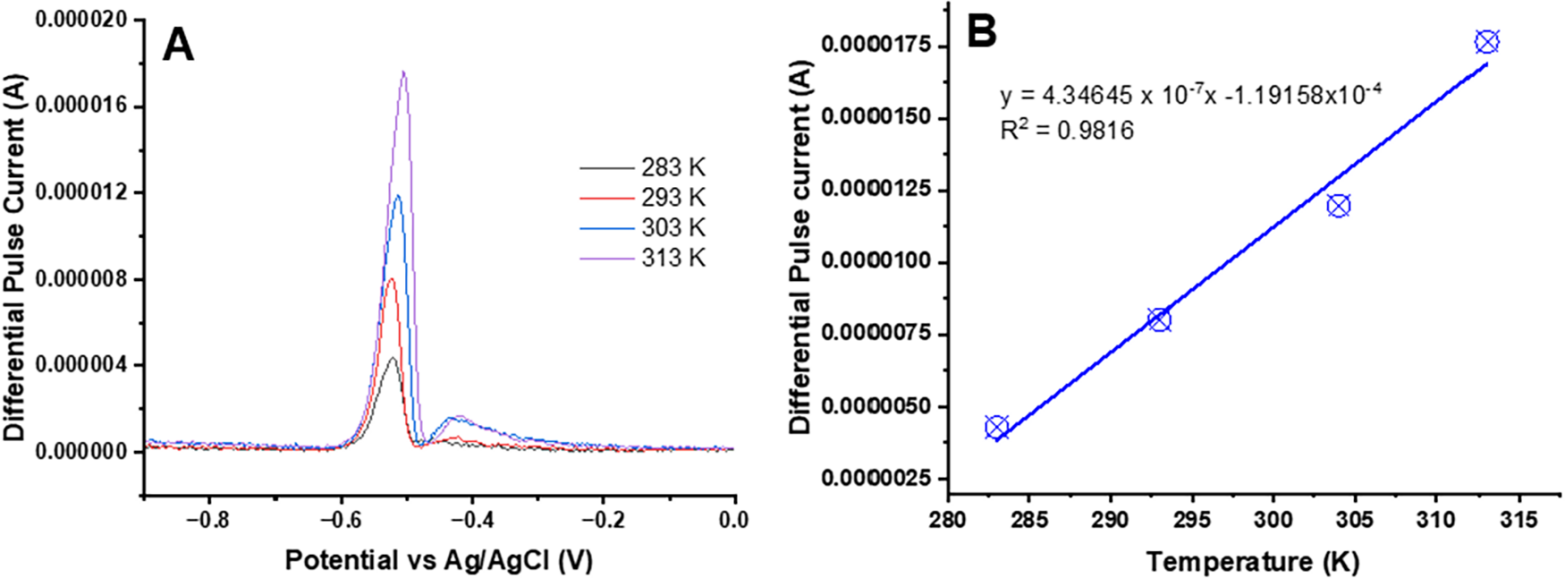
(A) Temperature-dependent DPASV graphs of UiO-66-NH_2_(Zr)-GO nanocomposite-coated GC electrode-based sensor devices for detecting Pb^2+^ ion in acetate buffer solution at various temperatures ranging from 283 to 313 K and (**B**) a linear calibration curve exhibiting the relationship between the output signal of the sensor and the temperature of the experiment. The experiment parameters were as follows: pH: 4.5; optimized deposition potential: −1.2 V (E vs Ag/AgCl); deposition time: 250 s.

### Interference Study

To evaluate the selectivity of the developed electrochemical sensor, interference experiments were performed to examine its response in the presence of common coexisting ions. The anti-interference capability of the fabricated UiO-66-NH_2_(Zr)-GO@GC sensor was examined using the DPASV method for the simultaneous detection of Cd^2+^, Pb^2+^, and Cu^2+^ ions in aqueous solutions. A range of common interfering ions, namely, Na^+^, K^+^, Ca^2+^, Mg^2+^, Fe^3+^, Mn^2+^, Ni^2+^, Zn^2+^, Hg^2+^, and Ag^+^, were introduced at concentrations five times higher than those of the target HMIs (10 μM). The results demonstrated that the presence of these interfering species, except Hg^2+^ exerted no significant influence on the electrochemical signals of Cd^2+^, Pb^2+^, or Cu^2+^. No additional or overlapping peaks were observed within the potential window of −1.0 to 0.2 V, indicating minimal electrochemical interference. Interestingly, when 5-fold higher [Hg^2+^] was introduced compared to the target HMIs (10 μM), it led to increases in the stripping currents of Pb^2+^ (∼7%), Cd^2+^ (∼9%), and Cu^2+^ (∼60%), as shown in Figure 13. In addition, a distinct mercury peak appeared at 0.35 V. This enhancement in stripping current is attributed to the partial reduction of Hg^2+^ during the preconcentration step, resulting in the formation of metallic Hg nanosites on the MOF-GO surface. These Hg sites likely facilitate metal nucleation and/or lower the overpotential for analyte metal deposition, thereby increasing stripping peak intensity. Similar signal enhancement phenomena have been reported for rGO/MoS_2_/CS/GCE electrodes, where Hg^2+^ addition promoted Pb^2+^ deposition through thin mercury film formation and intermetallic or hydride effects.^49^ Although full amalgam behavior is unlikely on solid MOF composites, this partial mercury activation mechanism plausibly accounts for the observed signal increases. Further work is warranted to confirm this interpretation. Overall, these results demonstrate that the UiO-66-NH_2_(Zr)–GO@GCE sensor exhibits high selectivity and reliable performance for the simultaneous detection of Cd^2+^, Pb^2+^, and Cu^2+^, even in complex aqueous environments containing high concentrations of foreign metal ions, excluding interfering factors such as inorganic compounds, suspended particles, and other components to better simulate actual water samples.

**Figure 13:**
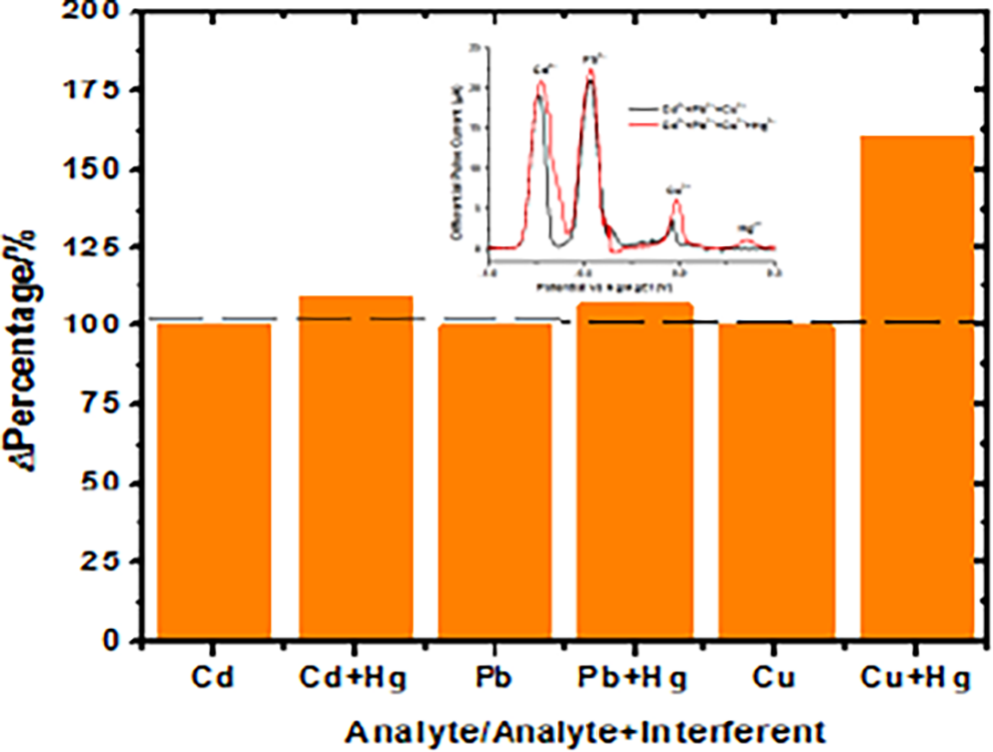
Interference study of 5-fold Hg^2+^ in the DPASV electrochemical determination of 10 μM solution of Cd^2+^, Pb^2+^, and Cu^2+^ in acetate buffer. The inset shows the DPASV curves for Cd^2+^, Pb^2+^, and Cu^2+^ in the presence and absence of Hg^2+^.

## Conclusions

In this study, a highly sensitive electrochemical sensor based on a UiO-66-NH_2_(Zr)-GO nanocomposite was successfully fabricated for the concurrent detection of multiple HMIs such as Cd^2+^, Pb^2+^, and Cu^2+^ ions in aqueous electrolyte solutions. The nanocomposite was synthesized via a one-pot hydrothermal method, resulting in a material with a high surface area, rich porosity, and abundant active adsorption sites, key attributes that significantly enhanced its sensing performance. The excellent sensitivity of the sensor is attributed to the synergistic interaction between UiO-66-NH_2_(Zr) and GO, which collectively improves electron-transfer kinetics and facilitates efficient heavy-metal ion adsorption. The fabricated sensor demonstrated excellent analytical performance, including a LOD of 7.5 nM for Cd^2+^, 14.1 nM for Pb^2+^, and 9.36 nM for Cu^2+^, all of which are well below the maximum residue levels recommended by the World Health Organization. These findings highlight the versatility and field relevance of the developed sensor as a reliable, high-sensitivity, and low-cost analytical tool for ppb-level detection of heavy metals in complex environmental systems, including natural waters, sediments, and wastewater.

## ∇

2305 Clairmont Rd NE, Atlanta, Georgia 30329, United States
